# Performance of Immunodiagnostic Tests for Typhoid Fever: A Systematic Review and Meta-Analysis

**DOI:** 10.3390/pathogens10091184

**Published:** 2021-09-13

**Authors:** Mohamad Ahmad Najib, Khairul Mohd Fadzli Mustaffa, Eugene Boon Beng Ong, Kasturi Selvam, Muhammad Fazli Khalid, Mohd Syafiq Awang, Nor Syafirah Zambry, Asrulnizam Abd Manaf, Yazmin Bustami, Hairul Hisham Hamzah, Asma Ismail, Ismail Aziah

**Affiliations:** 1 Institute for Research in Molecular Medicine (INFORMM), Universiti Sains Malaysia, Kubang Kerian 16150, Kelantan, Malaysia; najib@student.usm.my (M.A.N.); khairulmf@usm.my (K.M.F.M.); kasturiselvam0612@gmail.com (K.S.); fazlikhalid@usm.my (M.F.K.); norsyafirah@usm.my (N.S.Z.); asmainformm@yahoo.com (A.I.); 2 Institute for Research in Molecular Medicine (INFORMM), Universiti Sains Malaysia, Gelugor 11800, Pulau Pinang, Malaysia; eugene@usm.my; 3 Collaborative Microelectronic Design Excellence Centre (CEDEC), Universiti Sains Malaysia, Bayan Lepas 11900, Pulau Pinang, Malaysia; msa15_bio093@student.usm.my (M.S.A.); eeasrulnizam@usm.my (A.A.M.); 4 School of Biological Sciences, Universiti Sains Malaysia, Gelugor 11800, Pulau Pinang, Malaysia; ybustami@usm.my; 5 School of Chemical Sciences, Universiti Sains Malaysia, Gelugor 11800, Pulau Pinang, Malaysia; hishamhamzah@usm.my

**Keywords:** immunodiagnostic, typhoid, systematic review, sensitivity, specificity

## Abstract

Typhoid fever, also known as typhoid, is a life-threatening bacterial infection that remains a global health concern. The infection is associated with a significant morbidity and mortality rate, resulting in an urgent need for specific and rapid detection tests to aid prevention and management of the disease. The present review aims to assess the specificity and sensitivity of the available literature on the immunodiagnostics of typhoid fever. A literature search was conducted using three databases (PubMed, ProQuest and Scopus) and manual searches through the references of identified full texts to retrieve relevant literature published between 1 January 2011 and 31 December 2020. Of the 577 studies identified in our search, 12 were included in further analysis. Lipopolysaccharides (LPS) and hemolysin E (HlyE) were the most frequently studied antigens. The specimens examined in these studies included serum and saliva. Using blood culture as the gold standard, anti-LPS IgA gave the highest sensitivity of 96% (95% CI: 93–99) and specificity of 96% (95% CI: 93–99) for distinguishing between typhoid cases and healthy controls, whereas the combination of anti-LPS and anti-flagellin total IgGAM gave the highest sensitivity of 93% (95% CI: 86–99) and specificity of 95% (95% CI: 89–100) for distinguishing typhoid cases and other febrile infections. A comparably high sensitivity of 92% (95% CI: 86–98) and specificity of 89% (95% CI: 78–100) were shown in testing based on detection of the combination of anti-LPS (IgA and IgM) and anti-HlyE IgG as well as a slightly lower sensitivity of 91% (95% CI: 74–100) in the case of anti-50kDa IgA. Anti-50kDa IgM had the lowest sensitivity of 36% (95% CI: 6–65) against both healthy and febrile controls. The development of a rapid diagnostic test targeting antibodies against lipopolysaccharides combined with flagellin appeared to be a suitable approach for the rapid detection test of typhoid fever. Saliva is added benefit for rapid typhoid diagnosis since it is less invasive. As a result, further studies could be done to develop additional approaches for adopting such samples.

## 1. Introduction

Typhoid fever is a systemic infection associated with the Gram-negative and rod-shaped bacillus *Salmonella enterica* serovar Typhi (*S.* Typhi). The life-threatening disease has been a public health problem in developing and underdeveloped countries for generations. A systematic analysis by typhoid and paratyphoid collaborators estimated that 10.9 million cases of typhoid fever occurred globally in 2017, resulting in 116.8 thousand deaths [1]. There is a declining global trend in typhoid incidence over the last few years with respect to the number of cases per capita, particularly in most high-income countries, such as Australia, Japan, New Zealand, Singapore, South Korea and Taiwan and middle-income countries, such as Malaysia and Thailand [2]. However, in some countries such as Ghana, Malawi, Fiji, China, Indonesia, Cambodia and Iraq, typhoid incidence shows a steady increase from 1990 to 2014. These numbers are more than likely an underrepresentation of the true disease burden given that a large proportion of patients are treated on an outpatient basis or receive no treatment at all. In Malaysia, recent estimates suggest that approximately 0.58 to 1.42 cases of *S*. Typhi infection per 100,000 population were reported each year between 2011 and 2015 and most of the cases were due to travelers returning from an endemic region, migrants and food chains being contaminated by food handlers [3].

*Salmonella* Typhi is an obligate pathogen and humans are the only host for this bacterium. Humans acquire the infection through ingestion of water or food contaminated with *S*. Typhi. Typhoid fever is a great risk in areas that have low-quality potable water, non-hygienic living conditions and improper sanitation systems. Asymptomatic carriers play an important role in introducing contamination and disease transmission [2]. The disease causes a prolonged fever that can be as high as 103–104 °F (39–40 °C), fatigue, headache, nausea, constipation or diarrhea and abdominal pain, which develops within 6 to 30 days after infection [4]. Severe cases may lead to serious complications or even death due to intestinal perforation [5,6].

Improving the quality of drinking water supplies and education in better hygiene practices are likely the most effective measures towards typhoid elimination. However, these interventions cannot be promptly realized in the endemic areas of Africa and South Asia. Therefore, short-term control of typhoid is largely dependent on extensive vaccination programs and appropriate treatment, both of which rely on the application of rapid diagnostic tests (RDTs) that have robust performance. There are several commercially available RDTs for typhoid fever but finding a typhoid diagnostic assay with a high degree of sensitivity or specificity is challenging. The lack of such tests limits the disease burden assessments and may result in patients being misdiagnosed and receiving suboptimal therapy. Furthermore, with the global increase in multidrug-resistant *S*. Typhi, the demand for typhoid diagnostics is growing [7,8,9].

The laboratory diagnosis of typhoid fever currently relies on blood and stool cultures, which is considered the gold standard for diagnosis. However, this may pose a major challenge in resource-limited settings, such as areas with a lack of basic laboratory facilities for culturing purposes [10]. Furthermore, the culture method is time-consuming, requiring several days for the isolation and identification of the causative agents [11]. The Widal test, which is the current standard serological method for typhoid diagnosis, relies on O and H antigens that have a relatively low specificity due to cross-reactivity with other bacterial infections [12]. Thus, a rapid and sensitive assay for the detection of *S.* Typhi would help both in clinical diagnosis and in preventing the spread of disease [13]. In this context, development of a highly specific and sensitive immunodiagnostic test for the diagnosis of typhoid fever is an appropriate approach.

Several serology-based rapid diagnostic tests for typhoid fever are commercially available, such as Typhidot (Malaysia), TUBEX (Sweden) and Multi-Test Dip-S-Tics (USA). These commercial RDTs have been globally evaluated for their performance. Despite that, none of these tests yielded satisfactory results when validated in different endemic setups [14]. This is due to the consistency in sensitivity level in those studies resulting from false negative issue. Numerous antigens have been evaluated for their effectiveness in detecting *S.* Typhi, such as flagellin, hemolysin E (HlyE), YncE and cytolethal distending toxin subunit B (CdtB) [15,16,17]. However, due to the lack of thorough comparisons of the available data, the option of the best antigen for *S*. Typhi detection is yet to be determined. An understanding of the specificity and sensitivity of previously identified *S*. Typhi biomarkers is critical in providing vital information for the selection of an effective target and consequently establishing highly accurate diagnostic approaches for typhoid fever. Therefore, the present review focuses on the evaluation of the available evidence regarding the specificity and sensitivity of *S*. Typhi antigens. It is intended that our findings will serve as an informative resource for researchers aiming to develop an accurate laboratory diagnostic test for typhoid fever.

## 2. Results

**Figure 1:**
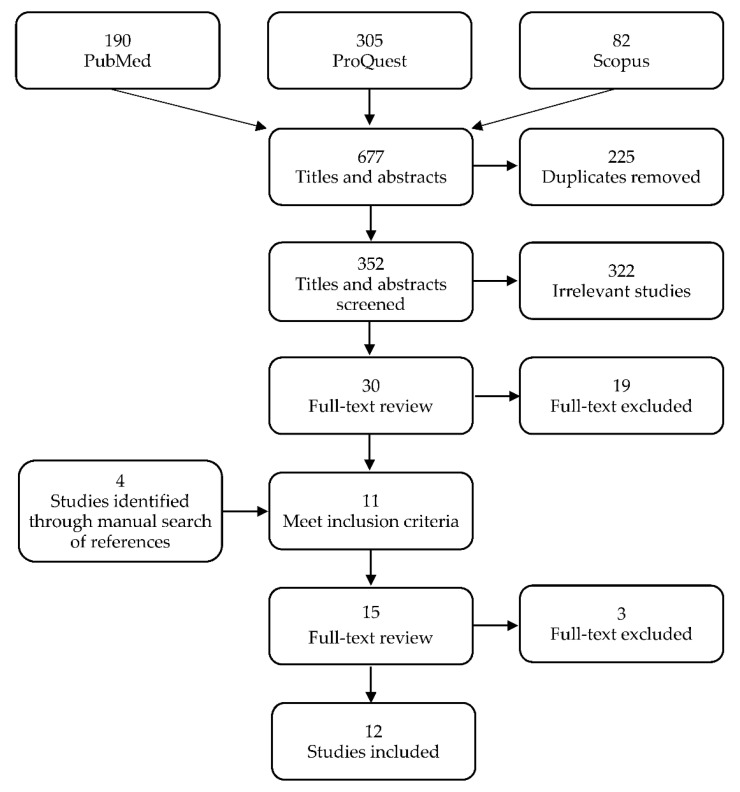
Preferred reporting items for systematic review and meta-analyses (PRISMA) flow diagram of search strategy and selection of studies.

Of the 577 studies that were identified from the three databases, 352 remained after 225 duplicates were removed. Of these, 322 were excluded during title and abstract screening and 19 were excluded during full-text screening based on the study criteria, leading to a total of 341 studies that were excluded. A total of four new studies were identified through manual searches of the lists of references and three were removed after the full-text screening. Twelve studies were included in the final review (Figure 1).

### 2.1. Characteristics of Studies

The study characteristics are summarized in Table 1. Overall, all 12 studies utilized antibody detection. A total of eight antigens were studied, which included lipopolysaccharides (LPS), hemolysin E (HlyE), flagellin, uncharacterized protein YncE, cytolethal distending toxin subunit B (CdtB), membrane proteins (MP), Vi antigen and 50 kDa outer membrane protein (OMP). LPS were the most frequently studied antigen, with 7 (58.3%) out of the 12 studies reporting on diagnostic sensitivity and specificity, followed by HlyE, which was reported in six (50.0%) studies. A total of eight (66.7%) studies evaluated the performance of antibody-based detection using ELISA, two (16.7%) studies performed detection using a lateral flow assay, one (8.3%) study used a dot enzyme immunoassay and one (8.3%) study used both ELISA and a lateral flow assay. Most of the studies tested for the presence of antibodies in serum sample and only two studies tested saliva.

### 2.2. Methodological Risk of Bias

**Figure 2:**
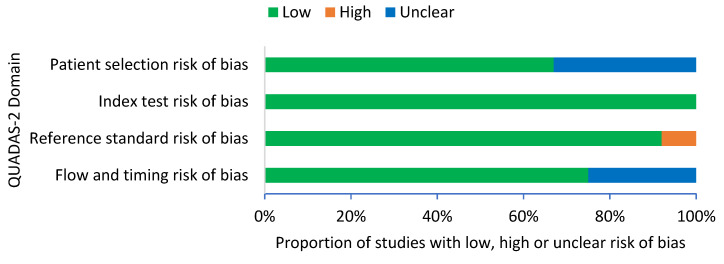
QUADAS-2 criteria among all studies (*n* = 12).

A summary of the QUADAS-2 assessment is presented in Figure 2. The overall results of the quality assessment showed a low risk of bias in all 12 studies. For patient selection, eight studies (67%) demonstrated a low risk of bias and four studies (33%) demonstrated an unclear risk of bias. For index test, all 12 studies (100%) demonstrated a low risk of bias with clear interpretation of the index test. For reference standard, 11 studies (92%) demonstrated a low risk of bias and 1 study (8%) demonstrated a high risk of bias, as the healthy controls were not confirmed with blood culture. For flow and timing, nine studies (75%) demonstrated a low risk of bias and three studies (25%) demonstrated an unclear risk of bias.

### 2.3. Performances of the Immunodiagnostic Assays

**Figure 3:**
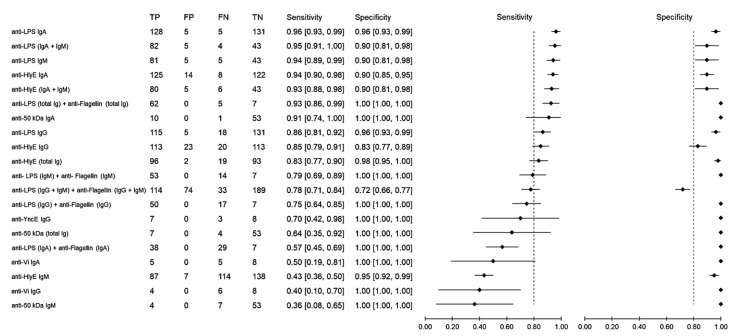
Meta-analysis of the diagnostic sensitivity and specificity of antibody detection tests with healthy individuals as a control group and blood culture as a reference standard.

Sensitivity measures the ability of a diagnostic test to correctly identify patients who have a disease with positive test results. Using samples from healthy individuals as the control group, the sensitivity of antibody detection varied between 36% and 96% (Figure 3). The anti-LPS IgA gave the highest sensitivity of 96% (95% CI: 93–99), followed by anti-LPS (IgA and IgM), which achieved a sensitivity of 95% (95% CI: 91–100), anti-LPS IgM with 94% (95% CI: 89–99) and anti-HlyE IgA with 94% (95% CI: 90–98). Specificity measures the ability of a diagnostic test to correctly identify people who do not have the disease with a negative test result. Comparably high specificities ranging from 72% to 100% were observed among all biomarkers. Only when the results of anti-LPS and anti-flagellin were combined did the specificity decrease to 72% (95% CI: 66–77), while all other results showed a specificity above 80%.

**Figure 4:**
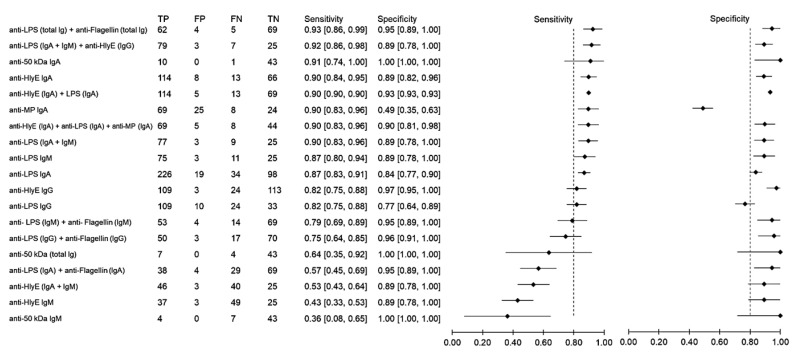
Meta-analysis of the diagnostic sensitivity and specificity of antibody detection tests with other febrile infections as a control group and blood culture as a reference standard.

Using samples from other febrile infections as the control group, the sensitivity and specificity of antibody detection tests for distinguishing typhoid and other febrile infections were 36–93% and 88–80%, respectively (Figure 4). The highest sensitivity was observed when the results of anti-LPS and anti-flagellin were combined. The three combinations anti-LPS (IgA + IgM) + anti-HlyE IgG, anti-HlyE (IgA) and anti-50 kDa (IgA) had comparably high sensitivities. The specificity of the immunodiagnostic tests ranged between 49% and 100%. Only two biomarkers showed a specificity lower than 80%, namely anti-membrane protein IgA and anti-LPS IgG.

Analysis of the studies based on the immunodiagnostic detection of saliva and serum samples showed that the tests had heterogeneous performance. A lower sensitivity (89.2%) was observed for studies detecting anti-LPS IgA in saliva when compared to serum (92%) [19,24]. On the contrary, anti-LPS IgA in saliva gave a higher specificity of 100% compared to that in serum of 92%. For studies detecting antibodies (IgGAM) against a 50 kDa antigen, the dot enzyme immunoassay showed 100% sensitivity and specificity when using serum but 90.9% sensitivity and 85.4% specificity when using saliva [22]. When Ig class-specific screening was performed, the study showed a higher sensitivity for IgA (90.9%) compared to IgG (72.7%) and IgM (72.7%) in saliva, but for serum, IgG (90.9%) had a higher degree of sensitivity compared to IgA (36.4%) and IgM (63.6%).

## 3. Discussion

Typhoid fever remains a public health problem with significant morbidity and mortality worldwide, particularly in underdeveloped and developing countries. Effective management of the disease depends on the performance and turnaround time of diagnostic tests. Although commercial point-of-care RDTs for enteric fever are available as alternatives to the current reference standard test of blood, stool, or bone marrow culture or the widely used Widal test, their diagnostic accuracy is still unsatisfactory [27]. The performance of currently available rapid diagnostics tests for typhoid fever, namely the Tubex TF and Typhidot tests, was moderate, with an average sensitivity of 78% and a specificity of 87% for Tubex TF and an average sensitivity and specificity of 78% and 77%, respectively, for Typhidot [28]. The present systematic review sought to evaluate the performance of newly developed immunodiagnostic tests for typhoid fever reported in the past 10 years to identify an alternative option for accurate laboratory testing.

The present study discovered an important gap in the immunodiagnostics for typhoid fever, namely, that, for all 12 studies included in the final review, diagnosis was based on the detection of antibodies rather than antigens. Tests conducted to detect antibody responses to *S.* Typhi infection have revolutionized typhoid diagnostics by significantly increasing detection capacity, allowing more people to be tested. However, such tests have limited utility because the tests are unable to produce rapid diagnosis in the case of acute infections, which hinders the process of taking immediate action and starting the needed course of treatment. This delay is due to the time required for the host immunological response to infection, for which detection is dependent, which can take several days [29]. Furthermore, the detection of antibodies does not always correlate with the existence of the bacteria, as antibodies can persist in the body for several months to years, making it challenging to distinguish between acute and convalescent cases [30,31]. The decay rates of IgG, IgA and IgM following *S*. Typhi infection were characterized in a kinetic study of human antibodies response after the onset of the infection [32]. The antibody decay profiles showed IgG antibodies persisted over 12 months after infection while the IgM and IgA antibodies appeared to decline after 3 to 4 months post infection. Another important limitation of antibody testing is that false positives may occur due to cross-reactivity resulting from other infections [33]. 

Detection of antigens, rather than antibodies, seems to be a suitable approach for the future development of RDTs for typhoid fever. A test that is able to detect *S.* Typhi antigens in clinical specimens could provide rapid and direct evidence of active disease. The lack of studies on antigen detection might reflect the difficulty in developing a reproducible system that can capture the target antigen in biological samples. However, there is an alternative to solve the problem, in which an aptamer can be used as the ligand instead of antibodies. Aptamers have unique advantages over antibodies as they can be produced in vitro, which reduces the potential for batch-to-batch variability and they are more stable and cheaper to produce, making them a suitable alternative to replace antibodies in antigen detection assays [34]. Even though antigen-based detection is superior to antibodies-based detection, but it has several limitations. First, antigen detection for typhoid fever cannot be performed using saliva as *S.* Typhi does not present in saliva during acute infection. Second, antigen-based detection is also challenging for carriers’ detection whereby intermittent shedding of the bacteria resulting in higher number of false negative cases. Therefore, combination of antigen-based tests can be a better alternative for carriers’ detection.

Anti-LPS was the most frequently studied antigen, probably because the use of *S.* Typhi O antigen for antibody detection in the Widal test has become the basis of antigen-based diagnostics. LPS are a component of the outer membrane and protect bacterial cells from the actions of the innate immune system during infection [35]. In addition, LPS are also the most abundant antigen on the cell surface of most Gram-negative bacteria, including *Salmonella* spp., making them a suitable target for typhoid testing. Another antigen that is frequently studied is HlyE, with all isotypes of antibodies having been evaluated. Since the first report of the presence of HlyE-reactive antibodies in typhoid patients in 2006 [36], several studies have shown that antibodies against the *S.* Typhi HlyE antigen are a promising biomarker for the detection of individuals with acute typhoid infections due to their ability to discriminate between typhoid cases and healthy individuals [37,38]. HlyE is a pore-forming cytotoxin of a 34 kDa protein that assembles into a ring-shaped dodeca-oligomer that forms stable pores in host membranes [39].

The detection system plays an important role in the process of developing RDTs. The advancement of RDT development, which is moving towards the utilization of label-free detection systems such as biosensors and paper-based sensors, shows good potential for future diagnostics [40]. However, the majority of the 12 studies still relied on ELISA, which requires labeling and advanced laboratory equipment such as a microplate reader, making its application in the field setting infeasible. Only three studies utilized a lateral flow assay as the detection system. This finding indicates the lack of point-of-care testing (POCT) development for typhoid fever. Adoption of POCT can help to reduce the turnaround times and to avoid sample transport problems, as onsite testing can be performed at the location of patientcare [41]. Therefore, there is a need to develop POCT for typhoid fever as rapid availability of results enables better clinical decisions, prevention and control, especially in rural areas where access to laboratory-based testing is not available. 

Non-invasive testing and sample collection are some of the important characteristics to be considered in the development of a new testing kit/method. Samples such as saliva and stool are quick and easy to collect and can be taken without the need for individual expertise. Our findings showed that only two studies used saliva samples. This may be partially attributed to the lack of studies on the saliva proteome. Most studies used serum as a test sample because blood is the most popular biological specimen used for laboratory diagnosis and the analysis of antibodies in serum has been widely reported [15,42]. The comparative performance of serum and saliva for the diagnosis of typhoid fever remains unclear. Detection of anti-LPS IgA in saliva gave a higher specificity but a lower sensitivity when compared to serum. Similarly, detection of specific Ig classes against a 50 kDa antigen showed a higher sensitivity for IgA when using saliva as compared to serum. However, detection of IgG showed a higher degree of sensitivity when using serum compared to saliva, while for IgM, saliva showed a higher sensitivity compared to serum. Combining all isotypes (IgGAM), serum showed a higher sensitivity and specificity compared to saliva. Therefore, further studies are needed to evaluate the performance of serum and saliva so that the best sample type can be determined for each biomarker.

Our meta-analysis showed that anti-LPS IgA gave the best diagnostic performance for distinguishing between typhoid cases and healthy individuals, with a sensitivity of 96% and a specificity of 96%. The IgA responses to LPS were also identified in previous typhoid microarray studies, in which the authors suggested including *S*. Typhi anti-LPS IgA in the development of new immunodiagnostic assays [43]. However, a lower sensitivity (87%) and specificity (84%) were observed in studies that involved other febrile infections as the control group. Since typhoid fever symptoms are similar to those of many other infectious diseases such as malaria and dengue [44,45], RDTs for typhoid fever would need to have a high sensitivity for distinguishing typhoid fever from other febrile infections. RDTs that are good at distinguishing between typhoid cases and healthy individuals might be valuable for epidemiological purposes, but their clinical utility will be restricted. Combining the results of anti-LPS and anti-flagellin total Ig showed the highest sensitivity for distinguishing typhoid cases and other febrile infections, making it the best biomarker for typhoid testing. RDTs with such performance would be helpful to enable faster clinical decision-making by avoiding false positive results due to other infections. However, it is important to note that the two studies on the anti-LPS and anti-flagellin included in this review did not include non-Typhoidal *Salmonella* species in the febrile control group. The two studies only included dengue, malaria, paratyphoid, brucellosis, rickettsiosis and leptospirosis [14,18]. Therefore, there is a possibility for cross reactivity of the anti-LPS and anti-flagellin with non-Typhoidal *Salmonella* species and other Enterobacteriaceae organisms. Further prospective evaluations with a larger sample size, more febrile controls and at different settings are still needed to provide conclusive data.

On the other hand, the sensitivity and specificity of anti-HlyE ELISA as a diagnostic tool for the detection of individuals with typhoid fever were comparable to those of anti-LPS, with a sensitivity and specificity above the threshold of 90% in both control groups, making it a suitable alternative option for accurate rapid diagnostic testing. We also found that two studies reported higher levels of anti-YncE in chronic carriers. A study in 2013 reported that 7 out of 10 chronic carriers had detectable anti-YncE IgG and IgA in the blood [20]. Another study identified nine individuals seropositive against anti-HlyE who showed higher levels of antibodies against YncE, suggesting that these individuals could be transient carriers of *S.* Typhi [16]. These two findings indicate that anti-YncE is a potential biomarker for typhoid carrier detection. Such a biomarker is important for the development of a diagnostic assay that can detect *Salmonella* carriers, as it would be a powerful tool to estimate true disease burden and potential of transmission [46]. Overall, the number of studies that have developed and evaluated the immunodiagnostic tests for typhoid in the past ten years is still limited. There is a need for more field evaluation studies to be performed in the future in order to provide a comprehensive overview of the immunodiagnostic performance, leading to the development of highly sensitive and specific RDTs for typhoid testing.

The present review has several limitations. First, the present study considered sensitivity and specificity but not predictive values. The prevalence of the disease among the study population has a significantly greater impact on predictive values than sensitivity and specificity, making it difficult to compare predictive values between studies [47]. Secondly, the studies on anti-YncE, anti-CdtB, anti-Vi and anti-50 kDa has small sample sizes, which might have affected the analysis of their diagnostic performance [48]. An insufficient sample size may result in poor diagnostic performance when there are false negative patients in the healthy control group. Thirdly, publication bias might have resulted in overestimation of some of the diagnostic performances. For example, studies with poor diagnostic performance are less likely to be published. Although only a small number of relevant primary studies are available, our search of multiple literature databases and the manual search of references in the retrieved literature should have helped to minimize the risk of publication bias in our review. Finally, the unknown sensitivity of blood culture is likely to have affected the analysis of immunodiagnostic performances. Blood culture is recognized to be an imperfect gold standard [49]. Owing to the poor sensitivity of the blood culture method, which is dependent on the abilities and knowledge of the laboratory staff, patients with a negative blood culture in the control group bear the risk of including undetected *Salmonella* Typhi cases [47]. Polymerase chain reaction (PCR) seems to be a more suitable method for use as the reference standard, as several studies have reported that PCR is more sensitive than the culture method [50,51].

## 4. Methods

The present systematic review utilized the preferred reporting items for systematic review and meta-analyses (PRISMA) guidelines.

### 4.1. Search Strategy

The literature search was conducted in January 2021 according to the modified preferred reporting items for systematic reviews and meta-analyses guidelines [52]. The search was conducted through three databases (PubMed, ProQuest and Scopus) using lists of keywords with reference to the expanded Medical Subject Headings (MeSH) thesaurus. These keywords were combined using the Boolean operators OR (within key concepts) and AND (between key concepts) as follows: [“*Salmonella* Typhi” OR “typhoid”] AND [“antigen” OR “antibody”] AND [“diagnosis” OR diagnostic”] AND [“specificity” OR “sensitivity”]. An additional search was conducted by manually screening the references of the retrieved literature.

### 4.2. Selection of Studies

Articles were excluded if (i) the studies were published before 1 January 2011, or after 31 December 2020; (ii) the studies were published in languages other than English or Malay; (iii) the studies did not mention the types of antigens used. We limit the studies to post 2011 (10 years) as the older literatures are not relevance to represent current diagnostic performance of the newly developed assays. Only studies that analyzed samples of at least two groups consisting of healthy controls and typhoid patients confirmed by positive blood culture were included in this systematic review. The retrieved literature was downloaded into Mendeley reference manager and duplicates were identified and removed. The references were distributed to four authors, who independently reviewed the titles and abstracts. A satisfactory agreement for the screening process was assessed between the reviewers. All four reviewers performed full-text screening and summarized the findings. Data from the selected sources were collated and summarized using a standard charting table consisting of six domains: (i) detection assays; (ii) biomarker; (iii) biological specimens; (iv) sample size; (v) specificity and sensitivity; (vi) year of publication.

### 4.3. Data Analysis

The number of true positives (TF), true negatives (TN), false positives (FP) and false negatives (FN) was independently retrieved from each article by three investigators and entered into an Excel datasheet. Discordant findings were assessed through discussion and when in doubt, the authors sought verification. Using blood culture as the reference standard, we calculated the sensitivity and specificity for each of the studied biomarker. Sensitivity was calculated by dividing the number of true positive outcomes with the sum of true positive and false negative outcomes. Specificity was calculated by dividing the number of true negative outcomes with the sum of true negative and false positive outcomes. Performance comparison of the immunodiagnostic assays was presented using forest plots based on control groups.

$$Sensitivity=\frac{\mathrm{TP}}{\mathrm{TP}+\mathrm{FN}}$$

$$Specificity=\frac{\mathrm{TN}}{\mathrm{TN}+\mathrm{FP}}$$

### 4.4. Assessment of the Risk of Bias

The risk of bias for all included studies was assessed using the Quality Assessment of Diagnostic Accuracy Studies 2 (QUADAS-2) tool [53]. The QUADAS-2 tool comprises four domains: patient selection, index test, reference standard and flow and timing (Table 2). Signaling questions were included to help the authors judge the risk of bias. The risk of bias was categorized as low, high, or unclear for each domain following the recommendations of the authors of the QUADAS checklist. The assessment was performed by three authors independently and disagreements among the authors were resolved by discussion.

## 5. Conclusions

The present systematic review provides an overview of the performance of immunodiagnostic tests for typhoid fever and found that tests based on anti-LPS in combination with anti-flagellin offered the best diagnostic performance for the diagnosis of typhoid fever and that anti-HlyE could be an alternative option with a performance comparable to that of anti-LPS. Our results highlight the limitations of the ongoing immunodiagnostic development and provide baseline information for future studies to select appropriate biomarkers in the development of RDTs for typhoid fever. Based on the good performance of anti-LPS and anti-HlyE, these two antigens appear to be suitable options for the development of future POCTs for typhoid fever. The currently low number of studies using saliva samples for detection suggests that more studies could explore the development of alternative approaches implementing such samples, which have the advantages of less invasive sample collection, which would help to boost accessibility and facilitate efficient testing in a community, especially during a disease outbreak and in areas where access to laboratory-based testing is not available.

## Figures and Tables

**Table 1:** Characteristics of included studies.

	Method	Biomarker	Sample	Sensitivity and Specificity	No of Samples	Ref.
1	ELISA	Anti-LPS (IgG, IgM and IgA) anti-flagellin (IgG, IgM and IgA)	Serum	Total Ig: 93% and 95% IgG: 75% and 55% IgM: 79% and 95% IgA: 57% and 96%	Positive S. Typhi blood culture (*n* = 67), Widal positive (98), febrile controls (*n* = 216) and healthy controls (*n* = 7).	[18]
2	ELISA	Anti-LPS IgA	Saliva	89.2% and 100%	Positive S. Typhi blood culture (*n* = 37), febrile controls (*n* = 30) and healthy controls (*n* = 30).	[19]
3	IC-LFT	Anti-LPS (IgG IgM) and anti-flagellin (IgG IgM)	Serum	68.8% and 71.1%	Positive S. Typhi blood culture (*n* = 80) and negative S. Typhi blood culture (*n* = 256).	[14]
4	ELISA	Anti-YncE (IgG) and anti-Vi (IgG and IgA)	Serum	YncE IgG: 70% and 100% Vi IgG: 40% and 100% Vi IgA: 50% and 97%	S. Typhi carriers (*n* = 10), acute typhoid fever cases (*n* = 8), Nepalese controls undergoing elective cholecystectomy with negative bile cultures (*n* = 8) and healthy Bangladeshis (*n* = 8).	[20]
5	ELISA	Anti-HlyE (IgG IgM and IgA)	Serum	70% and 100%	Positive S. Typhi blood culture (*n* = 50), positive S. Paratyphi A blood culture (*n* = 6), other febrile infections (*n* = 19) and healthy individuals (*n* = 25).	[21]
6	LFT	Anti-HlyE (IgG IgA) and anti-LPS (IgG IgA)	Serum	HlyE IgG: 91.5% and 80.0% (against healthy) HlyE IgA: 94.4% and 90.0% (against healthy) HlyE IgG: 78.7% and 100% (against other febrile infections) HlyE IgA: 66.7% and 80% (against other febrile infections) LPS IgG: 89.3% and 100% (against healthy) LPS IgA: 94.4% and 90.0% (against healthy) LPS IgG: 82.1% and 60.0% (against other febrile infections) LPS IgA: 81.8% and 54.5% (against other febrile infections)	Positive S. Typhi blood culture (*n* = 47), other febrile infections (*n* = 15), febrile with no bacterial growth (*n* = 67), healthy U.S. adults (*n* = 11) and healthy Nigerian children (*n* = 10).	[22]
7	ELISA	Anti-HlyE (IgG IgA IgM) and anti-LPS (IgG IgA IgM)	Serum	HlyE IgG: 84% and 90% (against Nigerian febrile controls) LPS IgA and IgM: 90% and 90% (against Nigerian febrile controls)	Nigerian pediatric typhoid cases (*n* = 86), Nigerian febrile controls (*n* = 28) and Nigerian healthy controls (*n* = 48).	[23]
8	Dot-EIA	Anti-50 kDa (IgG IgA IgM)	Saliva, serum	50 kDa IgGAM: 90.9% sensitivity and 85.4% (saliva) 50 kDa IgGAM: 100% and 100% (serum)	Positive S. Typhi blood culture (*n* = 11), non-typhoid fever patients (*n* = 43) and healthy (*n* = 53).	[24]
9	ELISA	Anti-CdtB IgM	Serum	100% and 83.3%	Positive S. Typhi blood culture (*n* = 21) and healthy controls (*n* = 12).	[25]
10	ELISA	Anti-HlyE IgA, anti-MP IgA and anti-LPS IgA	Serum	HlyE: 90% and 87% LPS: 90% and 77% MP: 90% and 48% HlyE and anti-LPS: 90% and 92% HlyE, LPS and MP: 90% and 90%	Positive S. Typhi blood culture (*n* = 105), healthy controls (*n* = 84) and other febrile disease (*n* = 64).	[15]
11	ELISA	Anti-HlyE (IgG IgA IgM) and anti-YncE (IgG IgA IgM)	Serum	HlyE IgGAM: 83% and 98%	Acute typhoid cases (*n* = 115), healthy controls (*n* = 95), other febrile infections (*n* = 95) and food handlers (*n* = 117).	[16]
12	ELISA, DDP-LF	Anti-LPS IgA and anti-HlyE IgA	Serum	92% and 94% (against all controls) 90% and 96% (against febrile endemic controls)	Positive S. Typhi blood culture (*n* = 30), positive S. Paratyphi A blood culture (*n* = 20), healthy endemic controls (*n* = 25) and febrile endemic controls (*n* = 25).	[26]

HlyE = hemolysin E; LPS = lipopolysaccharides; CdtB = cytolethal distending toxin subunit B; MP = membrane protein; Ig = immunoglobulin; YncE = uncharacterized protein; kDa = Kilodalton; Vi = virulence antigen; IC-LFT = immunochromatography lateral flow test; DPP-LF = dual-path platform lateral flow; Dot-EIA = dot enzyme immunoassay; ELISA = enzyme-linked immunosorbent assay.

**Table 2:** QUADAS-2 risk of bias assessment criteria.

Domains	Criteria for Low Risk Assessment
Patient selection	Patient enrolment strategy is specified and free of bias. A case—control design and inappropriate exclusions are avoided.
Index test	The index test results are interpreted without knowledge of the results of the reference standard. The conduct or interpretation of the index test does not introduce bias.
Reference standard	The reference standard correctly classifies the target condition. The reference standard results are interpreted without knowledge of the results of the index test. The reference standard, its conduct, or its interpretation do not introduce bias.
Flow and timing	There is an appropriate interval between the index test(s) and reference standard. All patients receive the same reference standard. All patients included in the analysis and patient flow do not introduce bias.

## Data Availability

Not applicable.

## References

[1] Stanaway J.D., Reiner R.C., Blacker B.F., Goldberg E.M., Khalil I.A., Troeger C.E., Andrews J.R., Bhutta Z.A., Crump J.A., Im J. (2019). The global burden of typhoid and paratyphoid fevers: A systematic analysis for the global burden of disease study 2017. Lancet Infect. Dis..

[2] Als D., Radhakrishnan A., Arora P., Gaffey M.F., Campisi S., Velummailum R., Zareef F., Bhutta Z.A. (2018). Global trends in typhoidal salmonellosis: A systematic review. Am. J. Trop. Med. Hyg..

[3] Muhammad E.N., Abdul Mutalip M.H., Hasim M.H., Paiwai F., Pan S., Mahmud M.A.F., Yeop N., Tee G.H., Senin A.A., Aris T. (2020). The burden of typhoid fever in Klang Valley, Malaysia, 2011–2015. BMC Infect. Dis..

[4] Gunn J.S., Marshall J.M., Baker S., Dongol S., Charles R.C., Ryan E.T. (2014). *Salmonella* chronic carriage: Epidemiology, diagnosis, and gallbladder persistence. Trends Microbiol..

[5] Contini S. (2017). Typhoid intestinal perforation in developing countries: Still unavoidable deaths?. World J. Gastroenterol..

[6] Dougan G., Baker S. (2014). Salmonella enterica serovar Typhi and the pathogenesis of typhoid fever. Annu. Rev. Microbiol..

[7] Katiyar A., Sharma P., Dahiya S., Singh H., Kapil A., Kaur P. (2020). Genomic profiling of antimicrobial resistance genes in clinical isolates of Salmonella Typhi from patients infected with typhoid fever in India. Sci. Rep..

[8] Godbole G., McCann N., Jones S.M., Dallman T.J., Brown M. (2019). Ceftriaxone-resistant Salmonella Typhi in a traveller returning from a mass gathering in Iraq. Lancet Infect. Dis..

[9] Qamar F.N., Yousafzai M.T., Khalid M., Kazi A.M., Lohana H., Karim S., Khan A., Hotwani A., Qureshi S., Kabir F. (2018). Outbreak investigation of ceftriaxone-resistant Salmonella enterica serotype Typhi and its risk factors among the general population in Hyderabad, Pakistan: A matched case-control study. Lancet Infect. Dis..

[10] Castonguay-Vanier J., Davong V., Bouthasavong L., Sengdetka D., Simmalavong M., Seupsavith A., Dance D.A.B., Baker S., Phuong T.L.T., Vongsouvath M. (2013). Evaluation of a simple blood culture amplification and antigen detection method for diagnosis of Salmonella enterica serovar Typhi bacteremia. J. Clin. Microbiol..

[11] Baker S., Favorov M., Dougan G. (2010). Searching for the elusive typhoid diagnostic. BMC Infect. Dis..

[12] Adhikari A., Rauniyar R., Raut P.P., Manandhar K.D., Gupta B.P. (2015). Evaluation of sensitivity and specificity of ELISA against widal test for typhoid diagnosis in endemic population of Kathmandu. BMC Infect. Dis..

[13] Abdullah J., Saffie N., Sjasri F.A.R., Husin A., Abdul-Rahman Z., Ismail A., Aziah I., Mohamed M. (2014). Rapid detection of Salmonella Typhi by loop-mediated isothermal amplification (LAMP) method. Braz. J. Microbiol..

[14] Das S., Rajendran K., Dutta P., Saha T.K., Dutta S. (2013). Validation of a new serology-based dipstick test for rapid diagnosis of typhoid fever. Diagn. Microbiol. Infect. Dis..

[15] Andrews J.R., Khanam F., Rahman N., Hossain M., Bogoch I.I., Vaidya K., Kelly M., Calderwood S.B., Bhuiyan T.R., Ryan E.T. (2019). Plasma immunoglobulin A responses against 2 Salmonella Typhi antigens identify patients with typhoid fever. Clin. Infect. Dis..

[16] Franklin F., Chong C.W., Chua L.H., Anthony A.A., Liew M.W.O., Aziah I., Ong E.B.B. (2020). Evaluation of Salmonella Typhi antigen YncE alongside HlyE for the detection of typhoid fever and its carriers. Med. Microbiol. Immunol..

[17] Mitra R., Bhan S., Nath G., Kumar N., Ali Z. (2013). Development of a novel rapid immunodiagnostic kit based on flagellar 40 kDa antigen epitope for the detection of typhoid fever in Indian patients. Sci. World J..

[18] Fadeel M.A., House B.L., Wasfy M.M., Klena J.D., Habashy E.E., Said M.M., Maksoud M.A., Rahman B.A., Pimentel G. (2011). Evaluation of a newly developed ELISA against widal, TUBEX-TF and Typhidot for typhoid fever surveillance. J. Infect. Dev. Ctries.

[19] Zaka-ur-Rab Z., Abqari S., Shahab T., Islam N., Shukla I. (2012). Evaluation of salivary anti-Salmonella typhi lipopolysaccharide IgA ELISA for serodiagnosis of typhoid fever in children. Arch. Dis. Child..

[20] Charles R.C., Sultana T., Alam M.M., Yu Y., Wu-Freeman Y., Bufano M.K., Rollins S.M., Tsai L., Harris J.B., LaRocque R.C. (2013). Identification of immunogenic *Salmonella enterica* serotype Typhi antigens expressed in chronic biliary carriers of *S.* Typhi in Kathmandu, Nepal. PLoS Negl. Trop. Dis..

[21] Ong E.B., Ignatius J., Anthony A.A., Aziah I., Ismail A., Lim T.S. (2015). Multi-isotype antibody responses against the multimeric Salmonella Typhi recombinant hemolysin E antigen. Microbiol. Immunol..

[22] Davies D.H., Jain A., Nakajima R., Liang L., Jasinskis A., Supnet M., Felgner P.L., Teng A., Pablo J., Molina D.M. (2016). Serodiagnosis of acute typhoid fever in Nigerian pediatric cases by detection of serum IgA and IgG against hemolysin E and Lipopolysaccharide. Am. J. Trop. Med. Hyg..

[23] Felgner J., Jain A., Nakajima R., Liang L., Jasinskas A., Gotuzzo E., Vinetz J.M., Miyajima F., Pirmohamed M., Hassan-Hanga F. (2017). Development of ELISAs for diagnosis of acute typhoid fever in Nigerian children. PLoS Negl. Trop. Dis..

[24] Mohd Redhuan N.E., Chin K.L., Adnan A.S., Ismail A., Balaram P., Phua K.K. (2017). Salivary anti-50 kDa antibodies as a useful biomarker for diagnosis of typhoid fever. J. Clin. Diagnostic. Res..

[25] Sharma T., Sharma C., Sankhyan A., Bedi S.P., Bhatnagar S., Khanna N., Gautam V., Sethi S., Vrati S., Tiwari A. (2018). Serodiagnostic evaluation of recombinant CdtB of S. Typhi as a potential candidate for acute typhoid. Immunol. Res..

[26] Kumar S., Nodoushani A., Khanam F., DeCruz A.T., Lambotte P., Scott R., Bogoch I.I., Vaidya K., Calderwood S.B., Bhuiyan T.R. (2020). Evaluation of a rapid point-of-care multiplex immunochromatographic assay for the diagnosis of enteric fever. Clin. Sci. Epidemiol..

[27] Maheshwari V., Kaore N.M., Ramnani V.K., Sarda S. (2016). A comparative evaluation of different diagnostic modalities in the diagnosis of typhoid fever using a composite reference standard: A tertiary hospital-based study in Central India. J. Clin. Diagnostic. Res..

[28] Wijedoru L., Mallett S., Parry C.M. (2017). Rapid diagnostic tests for typhoid and paratyphoid (enteric) fever. Cochrane Database Syst. Rev..

[29] Herath H.M.T.U. (2003). Early diagnosis of typhoid fever by the detection of salivary IgA. J. Clin. Pathol..

[30] Choo K.E., Davis T.M., Ismail A., Ong K.H. (1997). Longevity of antibody responses to a Salmonella typhi-specific outer membrane protein: Interpretation of a dot enzyme immunosorbent assay in an area of high typhoid fever endemicity. Am. J. Trop. Med. Hyg..

[31] Ismail A. (2000). New Advances in the Diagnosis of Typhoid and Detection of Typhoid Carriers. Malays. J. Med. Sci..

[32] Strid M.A., Dalby T., Mølbak K., Krogfelt K.A. (2007). Kinetics of the Human Antibody Response against Salmonella enterica Serovars Enteritidis and Typhimurium Determined by Lipopolysaccharide Enzyme-Linked Immunosorbent Assay. Clin. Vaccine Immunol..

[33] Bhatti A.B., Ali F., Satti S.A. (2015). Cross-reactivity of rapid Salmonella Typhi IgM immunoassay in dengue fever without co-existing infection. Cureus J. Med. Sci..

[34] Dunn M.R., Jimenez R.M., Chaput J.C. (2017). Analysis of aptamer discovery and technology. Nat. Rev. Chem..

[35] Kintz E., Heiss C., Black I., Donohue N., Brown N., Davies M.R., Azadi P., Baker S., Kaye P.M., Woude M.V.D. (2017). Salmonella enterica Serovar Typhi lipopolysaccharide O-antigen modification impact on serum resistance and antibody recognition. Infect. Immun..

[36] Von Rhein C., Hunfeld K., Ludwig A. (2006). Serologic evidence for effective production of cytolysin A in Salmonella enterica serovars Typhi and Paratyphi A during human infection. Infect. Immun..

[37] Liang L., Juarez S., Nga T.V.T., Dunstan S., Nakajima-Sasaki R., Huw Davies D., McSorley S., Baker S., Felgner P.L. (2013). Immune profiling with a Salmonella Typhi antigen microarray identifies new diagnostic biomarkers of human typhoid. Sci. Rep..

[38] Chin K.L., Redhuan N.E.M., Balaram P., Phua K.K., Ong E.B.B. (2016). Detection of salivary IgA antibodies against the HlyE antigen as a diagnosis of typhoid fever. J. Clin. Diagnostic. Res..

[39] Von Rhein C., Bauer S., López Sanjurjo E.J., Benz R., Goebel W., Ludwig A. (2009). ClyA cytolysin from Salmonella: Distribution within the genus, regulation of expression by SlyA, and pore-forming characteristics. Int. J. Med. Microbiol..

[40] Singh A., Verma H.N., Arora K. (2014). Surface plasmon resonance-based label-free detection of Salmonella using DNA self-assembly. Appl. Biochem. Biotechnol..

[41] Shephard M., Shephard A., Matthews S., Andrewartha K. (2020). The benefits and challenges of point-of-care testing in rural and remote primary care settings in Australia. Arch. Pathol. Lab. Med..

[42] Tran Vu Thieu N., Trinh Van T., Tran Tuan A., Klemm E.J., Nguyen Ngoc Minh C., Voong Vinh P., Pham Thanh D., Ho Ngoc Dan T., Pham Duc T., Langat P. (2017). An evaluation of purified Salmonella Typhi protein antigens for the serological diagnosis of acute typhoid fever. J. Infect..

[43] Darton T.C., Baker S., Randall A., Dongol S., Karkey A., Voysey M., Carter M.J., Jones C., Trappl K., Pablo J. (2017). Identification of novel serodiagnostic signatures of typhoid fever using a Salmonella proteome array. Front. Microbiol..

[44] Naveen Kumar C., Ponniah M., Srikumar R., Vijayakumar R., Chidambaram R., Jayalakshmi G., Prabhakar Reddy E., Manoharan A., Sai Ravi Kiran B. (2017). Incidence of dengue fever in febrile patients and co-infection with typhoid fever in South India. Ann. Med. Health Sci. Res..

[45] Onyido A.E., Ifeadi C.P., Umeanaeto P.U., Irikannu K.C., Aribodor D.N., Ezeanya L.C., Ugha C.N., Obiechina I.O. (2014). Co-Infection of malaria and typhoid fever in Ekwulumili Community Anambra State, Southeastern Nigeria. N. Y. Sci. J..

[46] Parry C.M., Wijedoru L., Arjyal A., Baker S. (2011). The utility of diagnostic tests for enteric fever in endemic locations. Expert Rev. Anti-Infect. Ther..

[47] Thriemer K., Ley B., Menten J., Jacobs J., Van Den Ende J. (2013). A systematic review and meta-analysis of the performance of two point of care typhoid fever tests, tubex TF and typhidot, in endemic countries. PLoS ONE.

[48] Bujang M.A., Adnan T.H. (2016). Requirements for minimum sample size for sensitivity and specificity analysis. J. Clin. Diagnostic. Res..

[49] Storey H.L., Huang Y., Crudder C., Golden A., De Los Santos T., Hawkins K. (2015). A meta-Analysis of typhoid diagnostic accuracy studies: A recommendation to adopt a standardized composite reference. PLoS ONE.

[50] Amalina Z.N., Khalid M.F., Rahman S.F., Ahmad M.N., Najib M.A., Ismail A., Aziah I. (2021). Nucleic acid-based lateral flow biosensor for Salmonella Typhi and Salmonella Paratyphi: A detection in stool samples of suspected carriers. Diagnostics.

[51] Maude R.R., de Jong H.K., Wijedoru L., Fukushima M., Ghose A., Samad R., Hossain M.A., Karim M.R., Faiz M.A., Parry C.M. (2015). The diagnostic accuracy of three rapid diagnostic tests for typhoid fever at Chittagong Medical College Hospital, Chittagong, Bangladesh. Trop. Med. Int. Health.

[52] Liberati A., Altman D.G., Tetzlaff J., Mulrow C., Gotzsche P.C., Ioannidis J.P.A., Clarke M., Devereaux P.J., Kleijnen J., Moher D. (2009). The PRISMA statement for reporting systematic reviews and meta-analyses of studies that evaluate healthcare interventions: Explanation and elaboration. BMJ.

[53] Whiting P.F., Rutjes A.W.S., Westwood M.E., Mallett S., Deeks J.J., Reitsma J.B., Leeflang M.M.G., Sterne J.A.C., Bossuyt P.M.M. (2011). QUADAS-2: A revised tool for the quality assessment of diagnostic accuracy studies. Ann. Intern. Med..

